# Ambulatory Phonation Monitoring With Wireless Microphones Based on the Speech Energy Envelope: Algorithm Development and Validation

**DOI:** 10.2196/16746

**Published:** 2020-12-03

**Authors:** Chi-Te Wang, Ji-Yan Han, Shih-Hau Fang, Ying-Hui Lai

**Affiliations:** 1 Department of Otolaryngology Head and Neck Surgery Far Eastern Memorial Hospital New Taipei City Taiwan; 2 Department of Electrical Engineering Yuan Ze University Taoyuan Taiwan; 3 Department of Special Education University of Taipei Taipei Taiwan; 4 Department of Biomedical Engineering National Yang-Ming University Taipei Taiwan; 5 Ministry of Science and Technology Joint Research Center for Artificial Intelligence Technology and All Vista Healthcare Taoyuan Taiwan

**Keywords:** voice disorder, speech envelope, phonation habits, background noise, noise reduction, adaptive threshold, dosimetry, phonotrauma

## Abstract

**Background:**

Voice disorders mainly result from chronic overuse or abuse, particularly in occupational voice users such as teachers. Previous studies proposed a contact microphone attached to the anterior neck for ambulatory voice monitoring; however, the inconvenience associated with taping and wiring, along with the lack of real-time processing, has limited its clinical application.

**Objective:**

This study aims to (1) propose an automatic speech detection system using wireless microphones for real-time ambulatory voice monitoring, (2) examine the detection accuracy under controlled environment and noisy conditions, and (3) report the results of the phonation ratio in practical scenarios.

**Methods:**

We designed an adaptive threshold function to detect the presence of speech based on the energy envelope. We invited 10 teachers to participate in this study and tested the performance of the proposed automatic speech detection system regarding detection accuracy and phonation ratio. Moreover, we investigated whether the unsupervised noise reduction algorithm (ie, log minimum mean square error) can overcome the influence of environmental noise in the proposed system.

**Results:**

The proposed system exhibited an average accuracy of speech detection of 89.9%, ranging from 81.0% (67,357/83,157 frames) to 95.0% (199,201/209,685 frames). Subsequent analyses revealed a phonation ratio between 44.0% (33,019/75,044 frames) and 78.0% (68,785/88,186 frames) during teaching sessions of 40-60 minutes; the durations of most of the phonation segments were less than 10 seconds. The presence of background noise reduced the accuracy of the automatic speech detection system, and an adjuvant noise reduction function could effectively improve the accuracy, especially under stable noise conditions.

**Conclusions:**

This study demonstrated an average detection accuracy of 89.9% in the proposed automatic speech detection system with wireless microphones. The preliminary results for the phonation ratio were comparable to those of previous studies. Although the wireless microphones are susceptible to background noise, an additional noise reduction function can alleviate this limitation. These results indicate that the proposed system can be applied for ambulatory voice monitoring in occupational voice users.

## Introduction

Human voice is produced via the periodic vibrations of vocal folds, driven by expiratory airflow. Cumulative voice loads and excessive vocal fold vibrations result in phonotraumatic injuries, such as vocal nodules and polyps [1]. The common symptoms of dysphonia (ie, phonation discomfort) include hoarseness, vocal fatigue, increased effort, and throat pain, which may limit the performance and long-term careers of occupational voice users [2]. Dysphonia also results in considerable financial losses for individuals and society [3,4]; the estimated annual cost associated with dysphonia is US $2.5 billion [5]. In addition, voice-related disorders significantly lower the quality of life in terms of physical functioning, general health, bodily pain, fatigue, and role limitation [6].

The most recognized risk for voice disorders is occupational voice overuse, commonly found in salespeople, industrial/factory workers, teachers, clergy, lecturers, and singers [6,7]. Among these occupations, the teaching profession has been significantly investigated by academic researchers [8-10]. In comparison to other occupations, teachers are more likely to report voice problems and the negative effects of dysphonia on their work performance [11]. Roy et al [2] reported that the prevalence of voice disorders was significantly higher in teachers (137/1243, 11.0%) in comparison to nonteachers (80/1288, 6.2%). The lifetime prevalence of dysphonia for teachers (717/1243, 57.7%) was also significantly higher than that for nonteachers (371/1288, 28.8%).

Voice therapy has been widely applied as the first-line treatment for voice disorders related to voice overuse or abuse [12,13]. By implementing multiple treatment strategies, voice therapy can effectively ameliorate the dysphonic symptoms, lower the phonation effort, and improve the voice quality [13]. However, one of the major challenges for voice therapy is the carryover of voicing techniques and habits taught during treatment sessions into their daily lives. To facilitate the maintenance of adequate phonation behavior, serial studies proposed the concept of ambulatory voice monitoring with promising results [14-16]. Most of these studies used a contact microphone or accelerometer attached to the anterior neck [17,18]. Subsequent studies demonstrated that this technology could significantly aid patients in controlling and tracking their vocal hyperfunction [19,20]. Another device for ambulatory voice monitoring was designed as a neck collar embedded with a contact microphone [21]. Although contact microphones and accelerometers can accurately detect phonation via the vibration of neck skin, the wiring and taping involved with these devices may cause discomfort in users. Furthermore, voice usage was mostly analyzed over a certain period with post hoc feedback [22], whereas real-time monitoring of the phonation ratio has not yet been reported.

To overcome the limitations in current devices, we propose a novel automatic speech detection system using a wireless microphone to capture acoustic signals from users, which can eliminate the discomfort associated with the wiring and taping of contact microphones. Our study hypothesizes that the speech energy envelope received via a wireless microphone can be used for ambulatory phonation monitoring. To examine this hypothesis, we designed this research with the following objectives: (1) to investigate the detection accuracy of speech, (2) to compare the measured phonation ratio and length of speech segments with those in existing literature, and (3) to examine the robustness of the noise reduction algorithm in simulated noisy conditions.

## Methods

### Overall Study Design

We proposed an automatic speech detection system using a wireless microphone for real-time ambulatory voice monitoring. We invited 10 teachers to participate in the pilot study. We designed an adaptive threshold (AT) function to detect the presence of speech based on the energy envelope. All participants were equipped with a wireless microphone during a teaching session (around 40-60 minutes) in a quiet classroom (background noise <55 dB sound pressure level [SPL]). We developed software for manually labeling the speech segments according to the time and frequency domains. We randomly selected 25 utterances (10 seconds each) from the recorded audio files to acquire the coefficients required for the AT function using a genetic algorithm (GA). Another 5 random utterances were used to test the accuracy of the automatic speech detection system using manually labeled data as the ground truth. We also mimicked scenarios of noisy backgrounds by mixing 4 different types of noise (at a signal-to-noise ratio [SNR] of 0, 3, and 5 dB) into the original recordings. An adjuvant noise reduction function using a log minimum mean square error (logMMSE) [23] algorithm was applied to counteract the influence on detection accuracy.

### Participants

We invited 10 teachers to participate in this study. This study was conducted at Far Eastern Memorial Hospital and National Yang-Ming University. The study protocol was approved by the Research Ethics Review Committee of Far Eastern Memorial Hospital (FEMH 108019-E). For the first period of study, we recruited 5 teachers from April to June 2019; for the second period, we recruited another 5 teachers from February to April 2020. Each teacher was provided with a wireless microphone during a regular teaching session of 40-60 minutes. The average background noise level was controlled under 55 dB SPL, established as the controlled environment test condition.

### Automatic Speech Detection System

#### Overview

**Figure 1:**
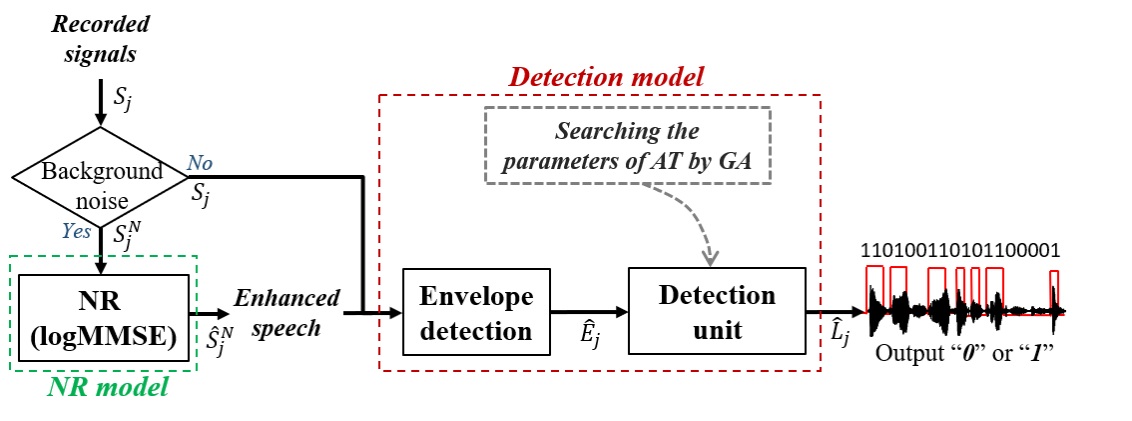
Proposed automatic speech detection system. AT: adaptive threshold; GA: genetic algorithm; logMMSE: logarithm minimum mean square error; NR: noise reduction.

Figure 1 illustrates the automatic speech detection system proposed in this study. The main corpus of this system is the detection model, which automatically divides acoustic signals into speech and nonspeech segments based on the energy envelope. We used a frame size of 32 milliseconds with a sampling rate of 16 kHz. Under simulated noisy conditions, the noise reduction model can be turned on to alleviate the effects from the background noise.

#### Detection Model

The signals (*S_j_*) recorded from the wireless microphone were converted to envelope (*Ê_j_*) by an “envelope detection” unit; the power energy can be used in this unit. Then, the “detection unit” predicted whether the input frames were speech or nonspeech by comparing the value of *Ê_j_* with that of the AT. The AT can be calculated using Equation (1); it is based on the energy of the input frame and 3 consecutive preceding frames.

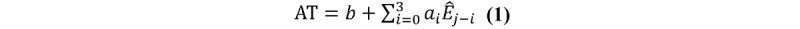



Here, *a_i_* represents the coefficients of the energy envelope of the current frame (*i*=0) and 3 successive preceding frames (*i*=1 to 3). It should be noted that the 3 successive preceding frames are included in this equation according to the best performance observed in our pilot study. *Ê_j-i_* represents the input acoustic energy features at the *j*-*i* frame index and *b* is the bias. When the value of *Ê_j_* exceeded the threshold derived from the AT in Equation (1), the system generated “*1*” as the output, indicating that this frame was recognized as speech. However, if the value was lower than the threshold derived from the AT in Equation (1), the system generated “*0*” (nonspeech) as the output.

**Figure 2:**
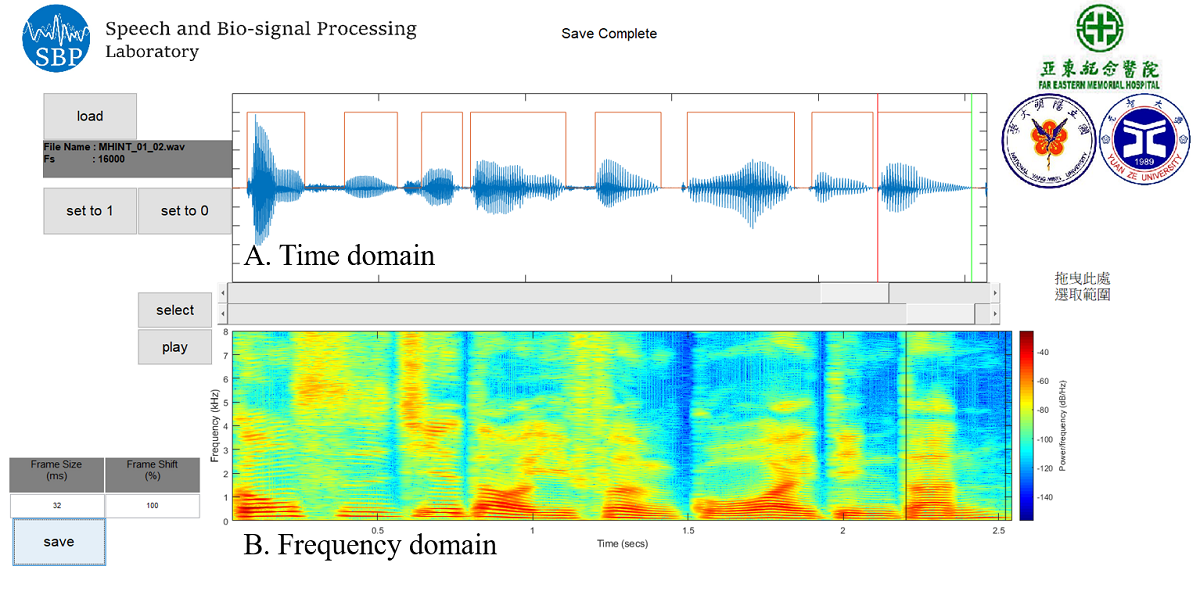
Overview of user interfaces in the proposed labeling tool. The selected speech segments are displayed as red brackets.

The 5 coefficients required to calculate the AT were defined by the following two steps: (1) manually labeling speech segments of the recoded audio files and (2) using a GA to search for these 5 coefficients. In the first step, we developed software (Figure 2) to manually label the speech segments according to their time and frequency domains. We applied the GA [24] to search for these 5 coefficients for the AT function based on 25 randomly selected utterances of 10 seconds each (details are provided in Multimedia Appendix 1). After acquiring the coefficients required for the AT function, another 5 random utterances were used to test the accuracy of the automatic speech detection system. Similarly, the manual labeling of the speech segments was considered as the ground truth. The overall accuracy of each subject was calculated based on each frame (ie, by dividing the predicted number of speech frames by the total number of speech frames labeled manually).

#### Noise Reduction Model

Because a wireless microphone is an air-conducted device that is susceptible to background noise, we performed additional experiments to examine the performance of the noise reduction model. We mimicked the presence of background noise by mixing the recorded speech signal (*S_j_*) with 4 different common background noises (crowd cheering noise, sharp speech noise, street noise, and white noise, shown in Multimedia Appendix 2) at 3 SNR levels (0, 3, and 5 dB), denoted by 
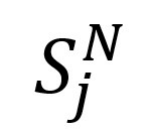
). The noisy signals (
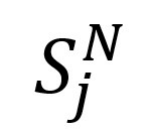
) were then processed by the logMMSE algorithm to obtain enhanced signals (
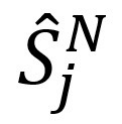
). Next, the 
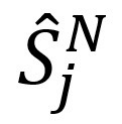
were sent into the detection model for energy and speech detection. We evaluated the performance of the noise reduction model by comparing the accuracy of speech detection under simulated noisy conditions with or without the noise reduction function.

#### Measuring Phonation Ratio and Duration of Speech Segments

To examine the applicability of the automatic speech detection system, we calculated the phonation ratio of the participants as shown in Equation (2), which is a common approach used to analyze phonation habits and usage [19,25].

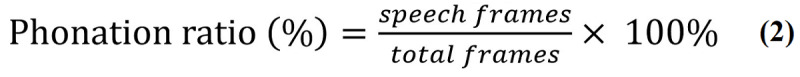



In addition, we calculated the duration and distribution of the phonation and nonphonation segments in a similar manner as in previous literature [22].

## Results

**Figure 3:**
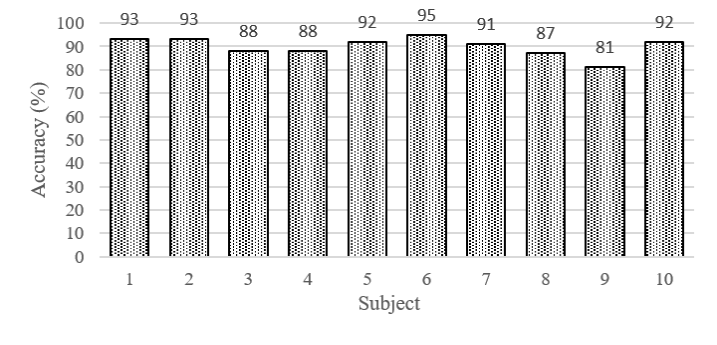
Average accuracy of the automatic speech detection system with respect to 10 teachers in a controlled environment.

**Figure 4:**
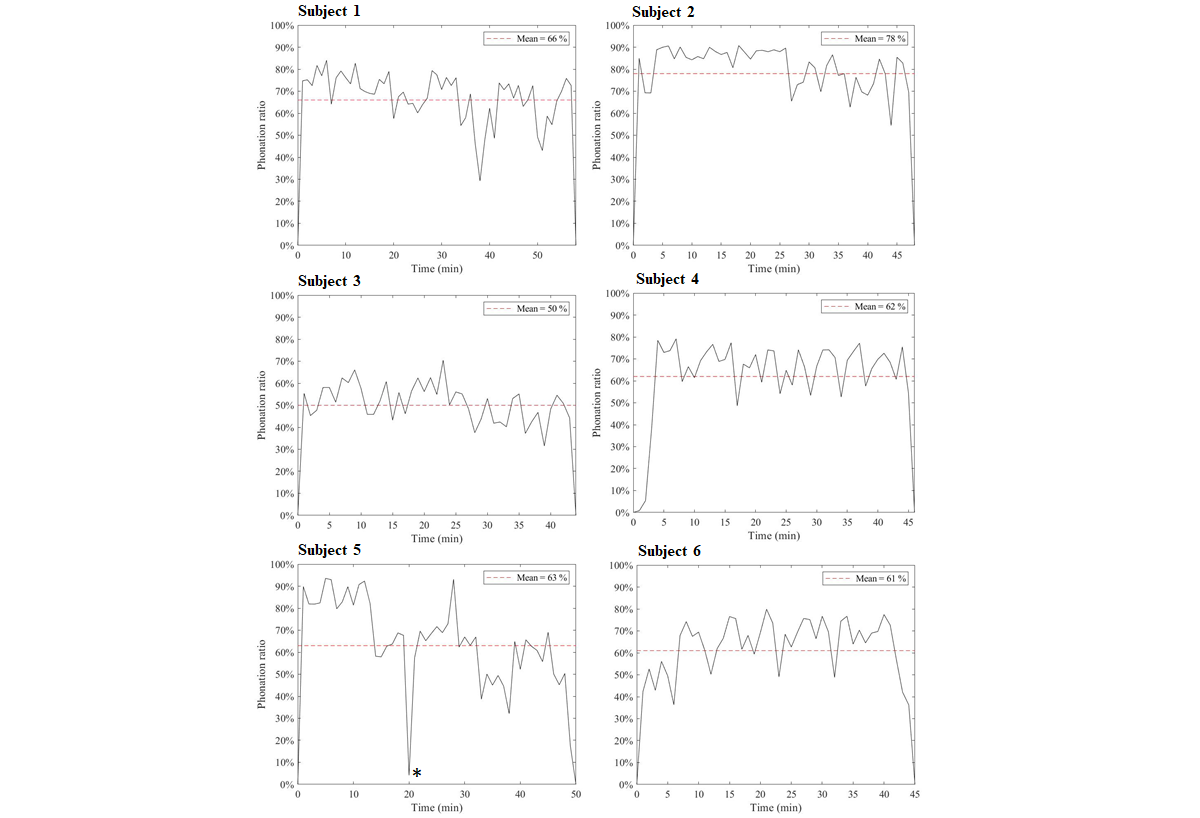
Phonation ratio over time for the 10 teachers (Subjects 1-6).

**Figure 5:**
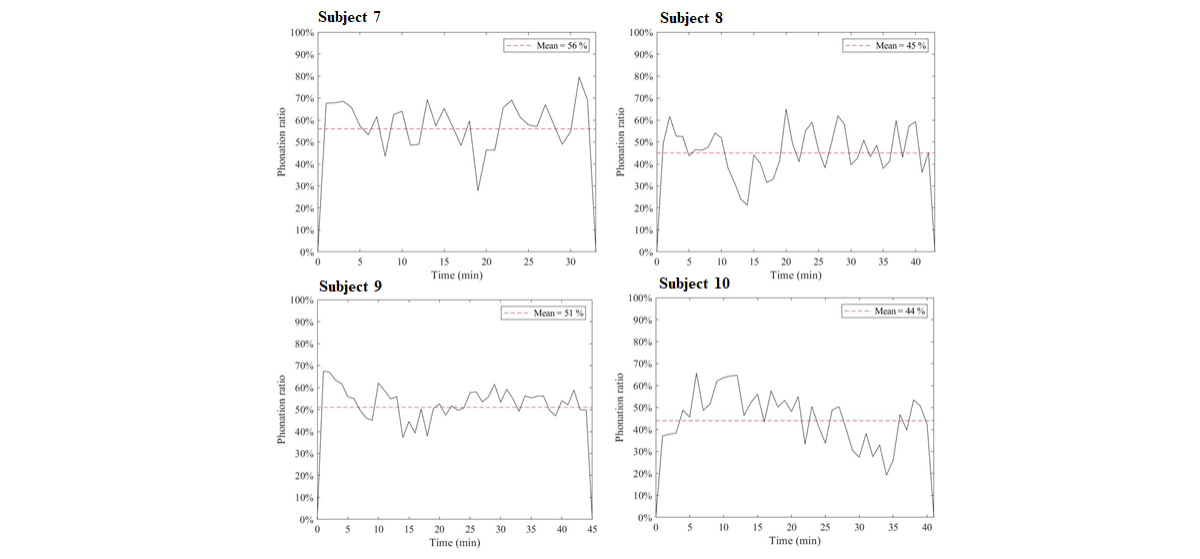
Phonation ratio over time for the 10 teachers (Subjects 7-10).

**Figure 6:**
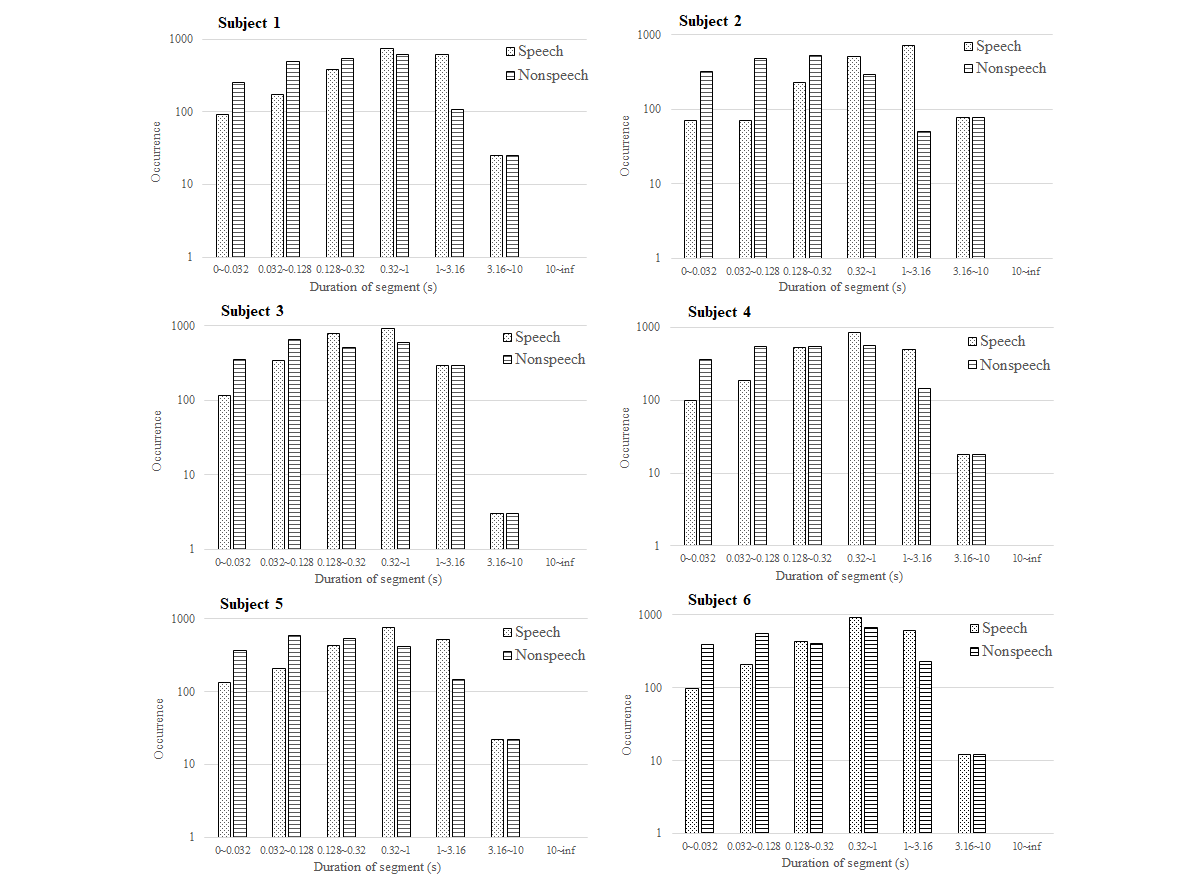
Speech and nonspeech segments measured by the automatic speech detection system. The x-axis indicates the length of the speech segments in a logarithmic scale, while the y-axis represents the occurrences during the recording period in a logarithmic scale (Subjects 1-6).

**Figure 7:**
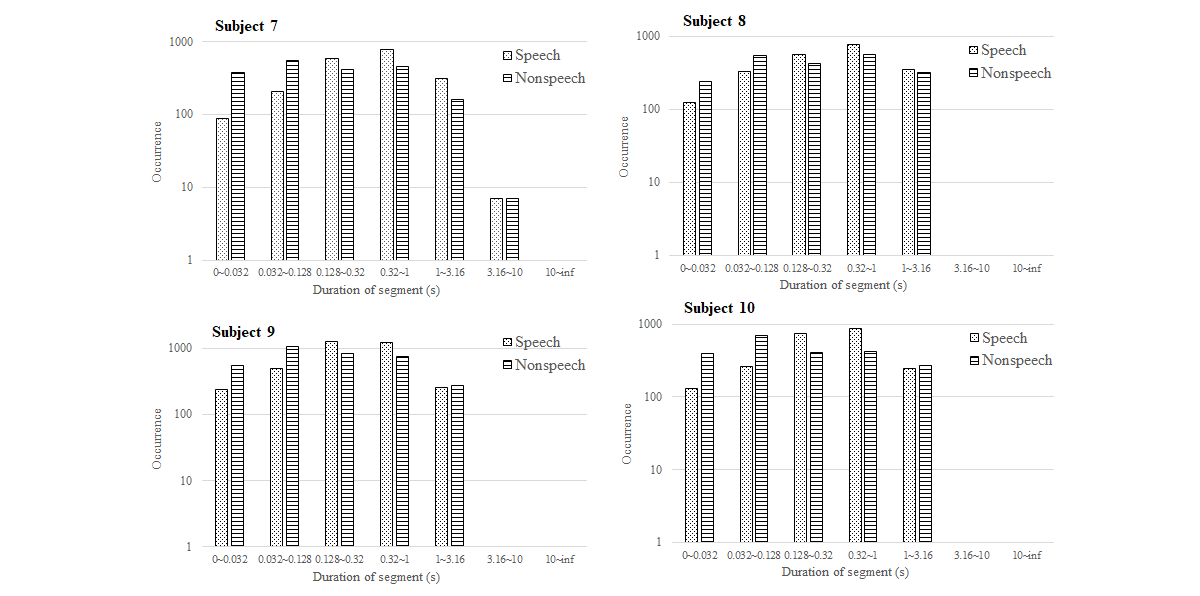
Speech and nonspeech segments measured by the automatic speech detection system. The x-axis indicates the length of the speech segments in a logarithmic scale, while the y-axis represents the occurrences during the recording period in a logarithmic scale (Subjects 7-10).

Figure 3 displays the average recognition accuracy of the automatic speech detection system, which was 89.9% (frame-based) in the controlled environment, ranging from 81.0% (67,357/83,157) to 95.0% (199,201/209,685). Figures 4 and 5 illustrate the phonation ratio for the 10 teachers evaluated during the teaching session. On average, the phonation ratio ranged from 44.0% (33,019/75,044) to 78.0% (68,785/88,186). We also noted a drastic decrease in the phonation ratio in subject 5 at approximately 20 minutes (asterisk, Figure 4). After reviewing the recorded audio file, we observed that this teacher did not speak for a while because he left the podium to fetch chalk; this example further demonstrated the excellent sensitivity of the proposed automatic speech detection system in practical scenarios. Figures 6 and 7 illustrate the distribution of the speech and nonspeech segments in logarithmic scales. Analytical results showed that the durations of most of the speech and nonspeech segments were less than 10 seconds.

**Figure 8:**
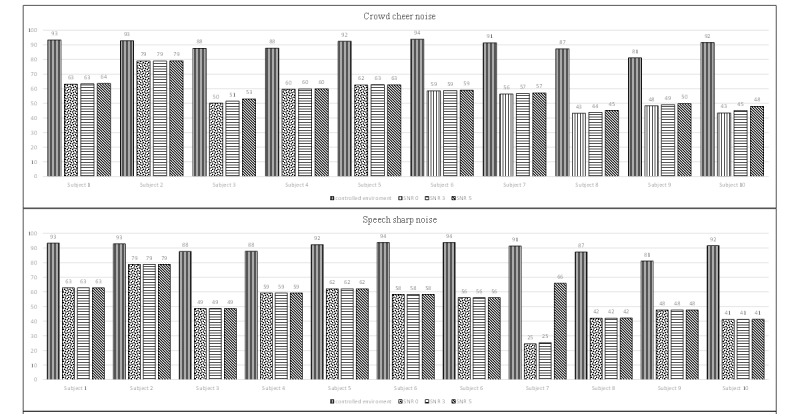
Accuracy of the automatic speech detection system with the presence of 2 different types of background noise (crowd cheer noise and speech sharp noise) at 3 SNR levels. The first bar of each graph indicates the accuracy of the original recording under the controlled environment. SNR: signal-to-noise ratio.

**Figure 9:**
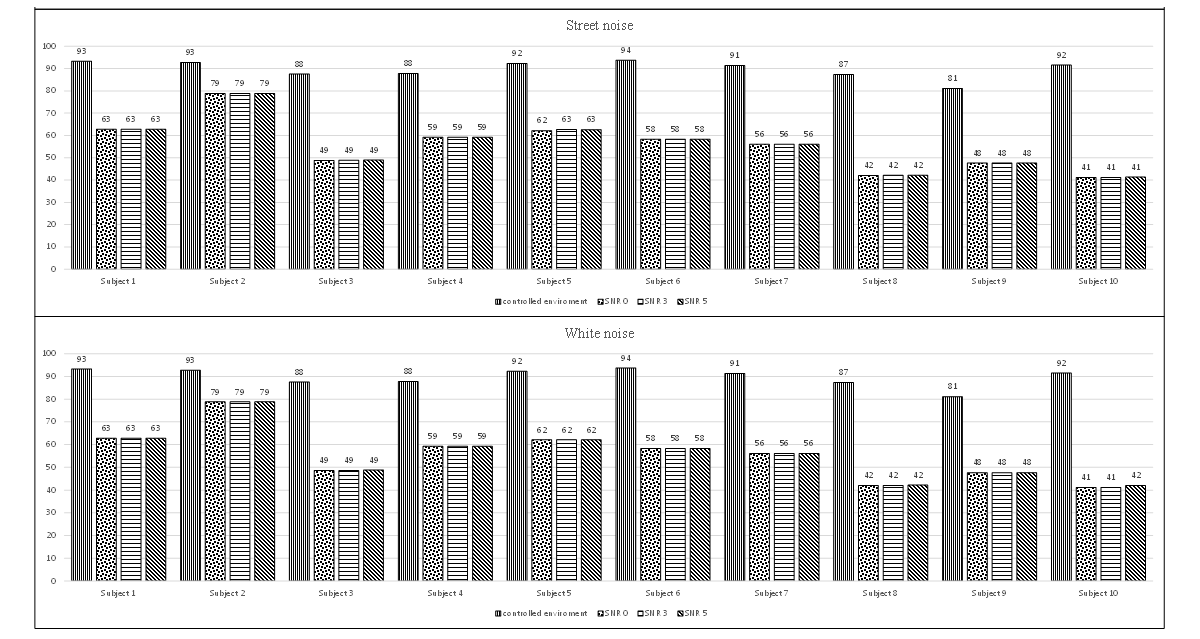
Accuracy of the automatic speech detection system with the presence of 2 different types of background noise (street noise and white noise) at 3 SNR levels. The first bar of each graph indicates the accuracy of the original recording under the controlled environment. SNR: signal-to-noise ratio.

Figures 8 and 9 present a comparison of the same recordings under the controlled environment and simulated noisy conditions. We noticed that the detection accuracy dropped significantly under noisy conditions, indicating that the performance of the automatic speech detection system can be easily affected by the presence of noise without a noise reduction function. On average, the additional noise decreased the accuracy by approximately 33%, 36%, 34%, and 36% for 4 different types of noise: crowd cheering noise, sharp speech noise, street noise, and white noise, respectively.

**Figure 10:**
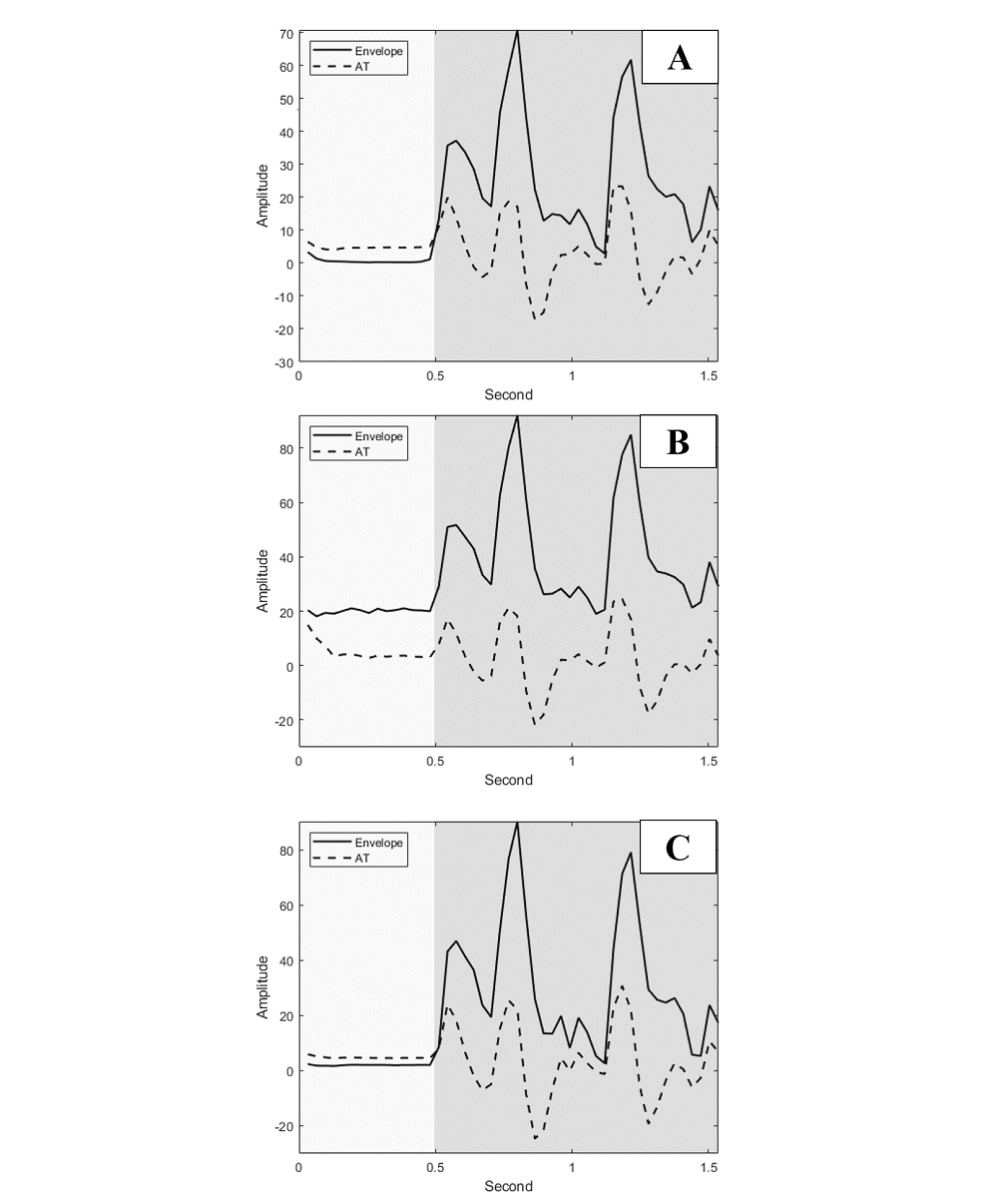
Example of the relationship between the speech envelope and AT in (A) the controlled environment, (B) noisy conditions without the noise reduction function, and (C) noisy conditions with the noise reduction function. Each part of the figure presents the same speech; the gray background denotes the speech segments, while the other areas indicate the nonspeech segments. AT: adaptive threshold.

Figure 10 presents an example of the relationship between the speech envelope and AT with and without the noise reduction function. Under the controlled environment, the AT was higher than the speech signal during nonspeech segments; in contrast, the energy envelope exceeded the AT in the presence of speech (Figure 10A). However, when the speech signal was contaminated by background noise, the overall energy exceeded the AT in both the speech and nonspeech segments (Figure 10B); thereby the proposed system may not be as effective in differentiating between speech and nonspeech signals. After enabling the logMMSE noise reduction function (Figure 10C), the AT could accurately detect the segments of speech versus nonspeech. On average, the additional noise reduction function yielded an average improvement of 4.9%, 27.5%, 19.3%, and 29.8% under the conditions of crowd cheering noise, sharp speech noise, street noise, and white noise, respectively. The detailed improvements are provided in Multimedia Appendix 3.

## Discussion

### Principal Findings

In this study, we proposed an ambulatory phonation monitoring system with a wireless microphone. The results demonstrated that the proposed system can accurately differentiate between speech and nonspeech segments based on the energy envelope in a controlled environment. The implementation of an additional noise reduction function using a logMMSE algorithm can effectively reduce the impact of background noise. Preliminary results of the phonation ratio and the distribution of speech segments in 10 teachers were compatible with those in previous literature [18,19].

### Applicability and Accuracy of Automatic Speech Detection System

Most studies in the existing literature used a neck accelerometer to detect the vibrations of vocal folds via skin [18,25]. Although contact microphones can effectively suppress the effects of background noise [16], they may not always be convenient for the users, owing to the cumbersome wiring and taping. In contrast, the wireless microphone used in this study eliminated the discomfort associated with the wiring and taping of contact microphones. All the participants reported good tolerance using wireless microphone, without any physical discomfort during the teaching session.

Previous studies [22] applied predefined criteria to detect voice activity, such as the fundamental frequency during normal speaking (ie, 70 to 1000 Hz), SPL greater than 30 dB, and a low/high ratio of at least 22 dB. In this study, we specifically designed software to manually label the speech segments (Figure 2), which served as the ground truth for examining the detection accuracy of this novel system. Figure 3 demonstrates an average detection accuracy of 89.9% in the controlled environment, which established the applicability and reliability of the proposed system.

In comparison to previous studies [18,25], our results demonstrated a higher phonation ratio (range: 44.0%-78.0%; Figure 3) owing to the continual lecturing of the teachers in the classroom. Similarly, the durations of most of the speech segments were less than 10 seconds. We did not observe long durations of silence (nonspeech) in this study (Figures 6 and 7). In contrast, previous studies recorded the phonation ratio throughout the day (except sleeping) [18,25]; thus, longer silence periods were more likely to be documented.

### Benefits of Noise Reduction Function

Because wireless microphones are more susceptible to background noise, we examined the effectiveness of the additional noise reduction function by mixing 4 different types of background noise to simulate noisy conditions. Our results showed that the noise reduction function using the logMMSE algorithm can improve the detection accuracy by up to 45.8% (maximum) in stable noise conditions (eg, sharp speech noise and white noise) (Multimedia Appendix 3); however, logMMSE works less efficiently in competing voice signals (eg, crowd cheering noise), resulting in an improvement of approximately 5%, similar to previous literature [26]. Accordingly, other noise reduction approaches, such as deep learning [27], may be more robust for enhancing the automatic speech detection system in the future. Additionally, automatic gain control [28] can also be integrated into the system to normalize the input volume and improve the accuracy in cases where sudden changes are observed in the input volume.

### Speaker Identification

The proposed system yielded comparable accuracy in most of the test conditions and an additional noise reduction function further improved the performance of the proposed system in noisy conditions. However, there is still room for improvement in some challenging conditions (eg, video sound or sudden increase in volume). We observed that subject 3 played a video clip with speech context during the class, and the loud speech from the video was misidentified as the speech of subject 3. For subject 4, several conversations took place between the teacher and students, which also caused the voice signals from the students to be misidentified as the speech of subject 4 and reduced the accuracy. One way to alleviate this inherent limitation of wireless microphones (ie, susceptibility to noise and competitive speakers) is the use of a microphone array with a beamforming algorithm that can fix (or adapt to adjust) the recorded position to distinguish between the speech of the speaker and background noise or other speakers. Another option to improve our system is implementing the speaker identification algorithm [29]; however, it requires significantly higher computing power to handle complex features (such as i-vector or x-vector [30,31]) using deep learning–based technology.

### Future Perspective

**Figure 11:**
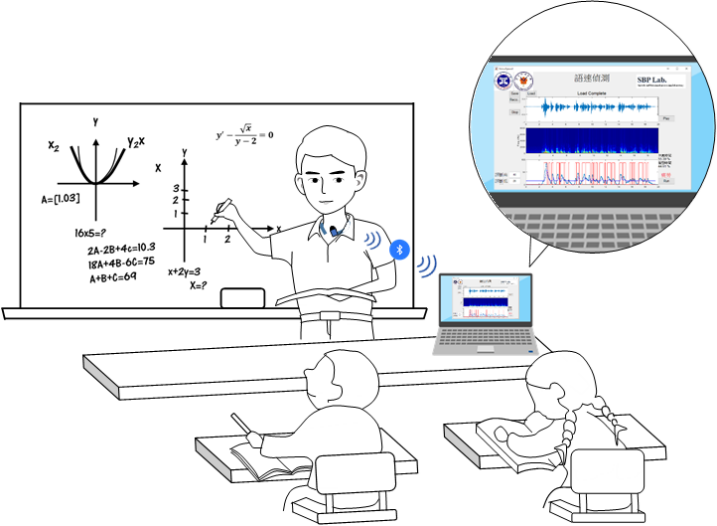
Exemplary use of the automatic speech detection system by a teacher.

The study results suggest that the proposed automatic speech detection system with wireless microphone can be applied in practical scenarios to overcome the limitations of contact microphone for ambulatory phonation monitoring. The proposed system can be further implemented on personal laptops (or mobile phone devices) for daily use and timely feedback, as illustrated in Figure 11. By monitoring the baseline phonation ratio, doctors and speech language pathologists can prescribe a certain threshold of phonation ratio based on individual conditions. Upon exceeding this limit, an alarm signal (flash or sound) could be sent to the user to ensure that they take enough breaks; promising results are available with respect to this concept [32] but it requires further evidential support from ongoing studies.

Although the proposed automatic speech detection system achieved 89.9% accuracy in this study for the proposed ambulatory phonation monitoring, it still has room for improvement. More recently, deep learning–based automatic speech recognition (ASR) [33] and natural language processing (NLP) [34,35] systems were proven to achieve higher speech recognition efficiency for conventional communication between human-machine applications (eg, Amazon Alexa, Google Home, and Apple Siri). These deep learning–based ASR and NLP systems could be applied in ambulatory phonation monitoring; however, some critical issues need to be addressed. For example, ASR and NLP technologies might violate the user’s privacy because they recognize the context of the user’s speech. In contrast, the automatic speech detection system of this study is energy-based; it will not directly access the content of speech and might be more acceptable to the users. In addition, ASR and NLP technologies require high computing power, especially when a deeper structure of the neural network is implanted to achieve higher speech recognition accuracy. A cloud-based ASR and NLP system could be effective in alleviating this limitation; however, the recorded speech data still needs to be uploaded to the server, which may lead to additional privacy and security issues. More recently, phonetic posteriorgram features obtained from the acoustic model of the ASR system was introduced for speech processing applications, and it has proven to achieve benefits in many tasks [36-38]. Following the success of phonetic posteriorgram, our future study could apply its features and deep learning technology to improve the performance of the current model.

Furthermore, this system can also be extended for detecting speech and communication disorders [39] (eg, Parkinson disease [40] and depression [41]). However, such work may require more sophisticated features of voice signals and computation techniques, such as the combination of the Mel frequency cepstral coefficients and deep neural networks, which was used in a previous study [42]. With the significant advancements in smartphones and smart home devices, the proposed automatic speech detection system can potentially be implemented in these devices to further decrease the clumsiness of any additional devices [43].

### Limitations

The first limitation of this study is the small number of participants (N=10). A larger cohort is required to obtain more robust evidence for the clinical use of automatic speech detection for ambulatory phonation monitoring. In addition, only teachers were recruited owing to the approved IRB protocol. Other occupations with high vocal demands (eg, salespeople and customer service representatives) will be included in the future to expand the potential use of the proposed system. Second, the proposed automatic speech detection system cannot precisely identify the speech of the speaker in the presence of loud competing background noise or other speakers. To overcome this issue, algorithms that require higher computing power, such as speaker identification or microphone array algorithms, could be used in future studies. Lastly, this automatic speech detection system requires manually labeling the recorded speech for model training. Considering the high accuracy achieved in this study, future research does not need to record the original voice content, so the confidentiality of the participants can be better protected.

### Conclusions

This study proposed an automatic speech detection system comprising a wireless microphone to receive the acoustic signals and an adaptive threshold for speech detection based on the energy envelope. The proposed system demonstrated a speech detection accuracy of 89.9%, and the analytical results for the phonation ratio and speech segments were comparable to those of previous research. Moreover, the use of an unsupervised noise reduction function (logMMSE) can improve the robustness of the proposed system in noisy conditions. These results imply that the proposed system can be a potential tool for ambulatory voice monitoring in occupational voice users.

## Appendix

Multimedia Appendix 1 Using genetic algorithm to determine the parameters of the adaptive threshold.

Multimedia Appendix 2 Spectrogram of the background noises used in this study.

Multimedia Appendix 3 Mean improvements in recognition accuracies when using the noise reduction function for the proposed auto speech detection system under simulated noisy conditions.

## Abbreviations

ASR: automatic speech recognition
AT: adaptive threshold
GA: genetic algorithm
logMMSE: log minimum mean square error
NLP: natural language processing
SPL: sound pressure level
