# Supplementary material for: Ambulatory Phonation Monitoring With Wireless Microphones Based on the Speech Energy Envelope: Algorithm Development and Validation
Source: JMIR Mhealth Uhealth. 2020 Dec 3;8(12):e16746. doi: 10.2196/16746 (PMC7746501; doi:10.2196/16746)
Supplement: Multimedia Appendix 1 [file mhealth_v8i12e16746_app1.docx]

## **Appendix 1. Using genetic algorithm to determine the parameters of the adaptive threshold.**

We randomly selected 25 utterances (of 10 s each) to determine the AT function with the GA, obtained from the ground truth dataset. As illustrated in Figure S2, we established 1,024 floating points ranging from -1 to 1 and encoded them into a 10-bit sequence. Accordingly, there were 50 bits in each chromosome, and 150 randomly initialized chromosomes were used to determine these five suitable parameters in Equation (1). Furthermore, a mutation rate of 10% was used for each generation in this study. Thereafter, the fitness function, as indicated in Equation (A1), was used to select suitable chromosomes from each generation. It should be noted that *n* defines the chromosome index, *J* represents the total number of frames, and c denotes the used data from the controlled environment. Moreover, $\hat{L}_{j}^{c}$ and $L_{j}^{c}$ are the predicted and target answers at the *j-th* frame index, respectively. A lower $L_{n}$ implied that the parameters in this generation were more suitable. Finally, the above steps were performed iteratively until the termination conditions, 300 generations, were satisfied. Table S1 presents the five parameters (four $a_{i}$ and one $b$) for the ten participants of this study, which were determined by the GA. From these searched results, it is clear that there is some difference between each user.

| $L_{n}={\sum_{j=1}^{J} \left\vert\hat{L}_{j}^{c}-L_{j}^{c} \right\vert}_{n}$ | (A1) |
| --- | --- |
|  |  |

Figure S2. Setting of the GA in this study.

Table S1. AT function parameters obtained by the GA. Column b denotes the function bias and $a_{0}$ represents the current frame, while $a_{1}$ to $a_{3}$ represent the three previous frames.

|  | $B$ | $a_{0}$ | $a_{1}$ | $a_{2}$ | $a_{3}$ |
| --- | --- | --- | --- | --- | --- |
| Subject 1 | 4.7890 | 0.2004 | -0.2199 | -0.2199 | -0.2199 |
| Subject 2 | 4.9316 | -1.0997 | -0.2297 | -0.2297 | -0.2297 |
| Subject 3 | 4.6774 | 0.9042 | -0.0440 | -0.0440 | -0.0440 |
| Subject 4 | 4.6285 | 0.6207 | -0.2395 | -0.2395 | -0.2395 |
| Subject 5 | 4.2864 | 0.4057 | -0.2102 | -0.2102 | -0.2102 |
| Subject 6 | 4.3744 | 0.3763 | -0.1711 | -0.1711 | -0.1711 |
| Subject 7 | 4.4819 | 0.6403 | -0.1515 | -0.1515 | -0.1515 |
| Subject 8 | 3.5142 | 0.855. | -0.0733 | -0.0733 | -0.0733 |
| Subject 9 | 4.8436 | 0.6794. | -0.0831 | -0.0831 | -0.0831 |
| Subject 10 | 4.7849 | 0.7869 | -0.0733 | -0.0733 | -0.0733 |
