# Supplementary material for: Ambulatory Phonation Monitoring With Wireless Microphones Based on the Speech Energy Envelope: Algorithm Development and Validation
Source: JMIR Mhealth Uhealth. 2020 Dec 3;8(12):e16746. doi: 10.2196/16746 (PMC7746501; doi:10.2196/16746)
Supplement: Multimedia Appendix 2 [file mhealth_v8i12e16746_app2.docx]

## **Appendix 2: Spectrogram of the background noises used in this study.**

Figure S3. Spectrogram of the four background noise signals used in this study: crowd cheer noise, speech sharp noise, street noise, and white noise.
