# Supplementary material for: Ambulatory Phonation Monitoring With Wireless Microphones Based on the Speech Energy Envelope: Algorithm Development and Validation
Source: JMIR Mhealth Uhealth. 2020 Dec 3;8(12):e16746. doi: 10.2196/16746 (PMC7746501; doi:10.2196/16746)
Supplement: Multimedia Appendix 3 [file mhealth_v8i12e16746_app3.docx]

## **Appendix 3.**

Table S2. Mean improvements in recognition accuracies when using the noise reduction function for the proposed auto speech detection system under simulated noisy conditions. A higher value indicates a better performance

| **Noise types** | **SNR level** | **Subjects** | | | | | | | | | | |  | |
| --- | --- | --- | --- | --- | --- | --- | --- | --- | --- | --- | --- | --- | --- | --- |
|  |  | ***1*** | ***2*** | ***3*** | ***4*** | ***5*** | ***6*** | ***7*** | ***8*** | ***9*** | ***10*** | | ***Ave.*** | |
| **Crowd cheer** | ***0 dB*** | 3.3 | 1.0 | 6.3 | 3.4 | 2.7 | 2.7 | 3.2 | 6.4 | 2.5 | 8.4 | **4.0** | |  |
|  | ***3 dB*** | 4.3 | 1.1 | 6.1 | 4.5 | 3.7 | 3.7 | 5.4 | 7.9 | 5.3 | 10.2 | **5.2** | |  |
|  | ***5 dB*** | 4.7 | 1.4 | 5.9 | 5.2 | 4.1 | 4.1 | 5.4 | 8.5 | 5.3 | 12.4 | **5.7** | |  |
| **Speech sharp noise** | ***0 dB*** | 19.1 | 3.3 | 31.2 | 19.3 | 26.1 | 10.7 | 26.1 | 39.2 | 25.4 | 45.4 | **24.6** | |  |
|  | ***3 dB*** | 23.5 | 9.4 | 34.9 | 22.4 | 27.1 | 30.6 | 26.1 | 40.0 | 26.0 | 45.7 | **28.6** | |  |
|  | ***5 dB*** | 25.5 | 10.5 | 36.5 | 24.2 | 27.6 | 31.3 | 26.4 | 39.9 | 26.3 | 45.7 | **29.4** | |  |
| **Street noise** | ***0 dB*** | 14.6 | 1.2 | 23.1 | 12.3 | 10.2 | 10.2 | 15.6 | 25.0 | 17.9 | 31.0 | **16.1** | |  |
|  | ***3 dB*** | 16.9 | 4.1 | 26.6 | 17.5 | 15.7 | 15.7 | 18.2 | 29.7 | 21.4 | 38.8 | **20.5** | |  |
|  | ***5 dB*** | 18.8 | 4.6 | 27.5 | 18.1 | 16.8 | 16.8 | 19.7 | 29.7 | 22.0 | 38.1 | **21.2** | |  |
| **White noise** | ***0 dB*** | 25.9 | 0.1 | 37.0 | 24.8 | 27.1 | 27.1 | 26.7 | 41.4 | 28.1 | 45.4 | **28.4** | |  |
|  | ***3 dB*** | 27.7 | 12.2 | 38.0 | 27.1 | 28.2 | 28.2 | 27.4 | 41.2 | 28.4 | 45.8 | **30.4** | |  |
|  | ***5 dB*** | 29.0 | 13.0 | 37.7 | 28.1 | 28.9 | 28.9 | 27.8 | 40.6 | 27.9 | 45.4 | **30.7** | |  |
